# RE: An intercultural perspective towards supporting antipsychotic medication adherence in clinical practice

**DOI:** 10.1192/bjb.2023.51

**Published:** 2023-08

**Authors:** Mustafa Alachkar

**Affiliations:** Mersey Care NHS Foundation Trust, Liverpool, UK. Email: mustafa.alachkar@nhs.net

## An intercultural perspective towards supporting antipsychotic medication adherence: is it all about medication?

I read with great interest Zacharia's Reflections article,^1^ which provides an interesting and informative intercultural perspective on antipsychotic medication adherence, with a focus on people from minority ethnic backgrounds in the UK. I strongly support the argument put forward by the author for a ‘relational/intercultural’ approach rather than a ‘cultural literacy’ approach, especially in view of the limitations of cultural competence training and the impossibility of becoming familiar with all the cultures that patients may come from, as highlighted in the article. I was, in fact, left wanting to hear more about this ‘relational/intercultural’ approach proposed by Zacharia, which seems more humble, genuine, curious and person-centred than learning more about someone's culture through diversity and equality training. It also puts the clinician and the patient on equal footing, where the clinician is there to also learn from and be educated by the patient, and it puts the relationship between the two at the centre of the work. I would have liked to hear more about what this approach looks like in practice and how it can be taught and incorporated into routine history-taking, for example. It goes without saying that such an approach would be useful in engaging patients from minority ethnic backgrounds in general, beyond the issue of medication adherence. Herein lies my main criticism of the article: namely its overemphasis, in my view, on medication adherence as if it were a goal in its own right, especially in the context of people from minority ethnic backgrounds. Zacharia, rightly, highlights the ‘need to think differently about how we support our ethnic minority patients’. The author does a very good job also at highlighting how explanatory models of illness that are accepted or adopted by ethnic minority patients may differ from Western explanatory models. He also shows commendable respect for ‘informal’ mental healthcare providers, such as faith healers and religious leaders, and he advocates engaging with them rather than ignoring them. However, the author's main focus seems to be on how we can use every method at our disposal to convince the patient to take medication. Zacharia's own cited figures show that 74% of patients discontinue antipsychotic medications after 18 months. This figure does not specify patients’ ethnicity or cultural background, raising the question of whether addressing this issue in people from ethnic minorities specifically is justified, especially when the evidence suggests that patients from minority ethnic backgrounds are often overmedicated and are far more likely to be offered medication (including depot medication) than to be offered psychotherapy, compared with their White counterparts.^2^ Furthermore, although the predominant understanding of the aetiology of mental illness in the West is a biomedical one, a large and growing number of clinicians, researchers and patients believe in more psychological and socioeconomic explanations, which are not too dissimilar to those adopted by some ethnic minority groups. These explanations seemed to be missing from the dichotomy that Zacharia has drawn between Western explanations of illness (portrayed here to be the biomedical explanations) and metaphysical explanations adopted by certain minority ethnic groups. I therefore worry that equating Western understanding of the aetiology of mental illness with the biomedical model risks perpetuating a narrow perspective that has sadly dominated Western psychiatry, to the detriment of patients. The reference to the therapeutic alliance in the article is highly appreciated. Here again, however, the author overemphasises, in my view, the use of good therapeutic alliance in the service of medication adherence, when a good therapeutic alliance is in itself a vehicle towards improvement, not necessarily mediated by medication adherence. In other words, a positive relationship between the patient and his care coordinator, for example, will help his recovery even when the care coordinator does not use this positive relationship to convince the patient to take his medication. The author hopes that having more foreign-born or foreign-trained doctors and medical leaders may help clinicians towards cultural competences. I share his hope. My own observation, however, is that foreign-born or foreign-trained doctors are often reluctant to bring up their own culture or consider its relevance to the clinical encounter with patients, for example in educational spaces (case presentations etc.), assuming perhaps that they are here to learn Western medicine and that they therefore have to put aside such crucial and defining aspects of their identity as culture, ethnicity and religion. Balint groups may be a space where junior doctors could be encouraged to bring up issues of race, ethnicity and culture more freely and without fear of judgement. Finally, it is refreshing to see Zacharia advocating the incorporation of the patient's spiritual needs into the role of mental health practitioners. He goes on to suggest, quite rightly, that ‘we must be ethnocentric in the mental health field’, and that ‘care may therefore require culturally appropriate framing. This may include spiritual or sociocultural interventions alongside antipsychotics’. Alas, the final quote appears only in the conclusion, right at the end of an article the focus of which has been on how to make patients from minority ethnic backgrounds take medication, and more medication.
